# *S*(+)-(2*E*)-*N*-(2-Hydroxypropyl)-3-Phenylprop-2-Enamide (KM-568): A Novel Cinnamamide Derivative with Anticonvulsant Activity in Animal Models of Seizures and Epilepsy

**DOI:** 10.3390/ijms21124372

**Published:** 2020-06-19

**Authors:** Agnieszka Gunia-Krzyżak, Ewa Żesławska, Karolina Słoczyńska, Dorota Żelaszczyk, Aleksandra Sowa, Paulina Koczurkiewicz-Adamczyk, Justyna Popiół, Wojciech Nitek, Elżbieta Pękala, Henryk Marona

**Affiliations:** 1 Jagiellonian University Medical College, Faculty of Pharmacy, Chair of Organic Chemistry, Department of Bioorganic Chemistry, Medyczna 9, 30-688 Kraków, Poland; dorota.zelaszczyk@uj.edu.pl (D.Ż.); henryk.marona@uj.edu.pl (H.M.); 2 Pedagogical University, Institute of Biology, Podchorążych 2, 30-084 Kraków, Poland; ewa.zeslawska@up.krakow.pl; 3 Jagiellonian University Medical College, Faculty of Pharmacy, Department of Pharmaceutical Biochemistry, Medyczna 9, 30-688 Kraków, Poland; karolina.sloczynska@uj.edu.pl (K.S.); ola.charchut@gmail.com (A.S.); paulina.koczurkiewicz@uj.edu.pl (P.K.-A.); justyna.popiol@uj.edu.pl (J.P.); elzbieta.pekala@uj.edu.pl (E.P.); 4 Jagiellonian University, Faculty of Chemistry, Gronostajowa 2, 30-387 Kraków, Poland; wojciech.nitek@uj.edu.pl

**Keywords:** anticonvulsant, antiseizure, cinnamamide derivatives, crystallography, drug development, epilepsy, preclinical safety evaluation

## Abstract

Epilepsy is one of the most frequent neurological disorders affecting about 1% of the world’s human population. Despite availability of multiple treatment options including antiseizure drugs, it is estimated that about 30% of seizures still remain resistant to pharmacotherapy. Searching for new antiseizure and antiepileptic agents constitutes an important issue within modern medicinal chemistry. Cinnamamide derivatives were identified in preclinical as well as clinical studies as important drug candidates for the treatment of epilepsy. The cinnamamide derivative presented here: *S*(+)-(2*E*)-*N*-(2-hydroxypropyl)-3-phenylprop-2-enamide (*S*(+)-*N*-(2-hydroxypropyl)cinnamamide, compound KM-568) showed anticonvulsant activity in several models of epilepsy and seizures in mice and rats. It was active in a genetic animal model of epilepsy (Frings audiogenic seizure-susceptible mouse model, ED_50_ = 13.21 mg/kg, *i.p*.), acute seizures induced electrically (maximal electroshock test ED_50_ = 44.46 mg/kg mice *i.p*., ED_50_ = 86.6 mg/kg mice *p.o*., ED_50_ = 27.58 mg/kg rats *i.p*., ED_50_ = 30.81 mg/kg rats *p.o*., 6-Hz psychomotor seizure model 32 mA ED_50_ = 71.55 mg/kg mice *i.p*., 44 mA ED_50_ = 114.4 mg/kg mice *i.p*.), chronic seizures induced electrically (corneal kindled mouse model ED_50_ = 79.17 mg/kg *i.p*., hippocampal kindled rat model ED_50_ = 24.21 mg/kg *i.p*., lamotrigine-resistant amygdala kindled seizure model in rats ED_50_ = 58.59 mg/kg *i.p*.), acute seizures induced chemically (subcutaneous metrazol seizure threshold test ED_50_ = 104.29 mg/kg mice *i.p*., ED_50_ = 107.27 mg/kg mice *p.o*., ED_50_ = 41.72 mg/kg rats *i.p*., seizures induced by picrotoxin in mice ED_50_ = 94.11 mg/kg *i.p*.) and the pilocarpine-induced status epilepticus model in rats (ED_50_ = 279.45 mg/kg *i.p*., ED_97_ = 498.2 mg/kg *i.p*.). The chemical structure of the compound including configuration of the chiral center was confirmed by NMR spectroscopy, LC/MS spectroscopy, elemental analysis, and crystallography. Compound KM-568 was identified as a moderately stable derivative in an in vitro mouse liver microsome system. According to the Ames microplate format mutagenicity assay performed, KM-568 was not a base substitution or frameshift mutagen. Cytotoxicity evaluation in two cell lines (HepG2 and H9c2) proved the safety of the compound in concentrations up to 100 µM. Based on the results of anticonvulsant activity and safety profile, *S*(+)-(2*E*)-*N*-(2-hydroxypropyl)-3-phenylprop-2-enamide could be proposed as a new lead compound for further preclinical studies on novel treatment options for epilepsy.

## 1. Introduction

Epilepsy is characterized by recurrent unprovoked epileptic seizures and is recognized as an important cause of morbidity and mortality. It is estimated to affect about 1% of the world’s human population [1]. Pharmacotherapy is one of the treatment options for epilepsy. It requires usage of antiseizure drugs (ASDs). These drugs are also known as antiepileptic drugs (AEDs), but the name was recently changed as the drugs most likely prevent seizures rather than epileptogenesis [2]. Despite the availability of more than 30 antiseizure drugs, some patients still do not receive adequate seizure control [3,4]. In general, some seizures are defined as resistant when they do not respond to available drugs. In clinical practice, pharmacoresistance is recognized if it is not possible to achieve sustained seizure freedom with the use of two well tolerated and appropriately chosen antiseizure drugs [5]. Recent findings confirmed that the increased risk for drug resistant epilepsy is caused by age at onset, symptomatic epilepsy, abnormal neuroimaging findings, abnormal electroencephalography results, history of mental retardation, neuropsychiatric disorders, febrile seizure, and status epilepticus [6]. Antiseizure drugs are known to cause several adverse side effects including both Type A—pharmacology related and Type B—idiosyncratic ones. Among them, somnolence, dizziness, fatigue, cognitive dysfunction, mood changes, endocrine disorders, skin rashes, agranulocytosis, and liver toxicity are described [7]. Moreover, cosmetic side effects were also recognized and included acne, gingival hyperplasia, hair loss, hirsutism, and weight gain [8]. In order to overcome those issues, searching for novel, more efficient, and safer antiseizure and antiepileptic drugs has been of interest for researchers all over the world.

Cinnamamide derivatives have been effectively tested for anticonvulsant activity. It is worth noting that *N*-(3-aryl-2-propenoyl)amido moiety was proposed as a pharmacophore determining anticonvulsant properties [9]. Among cinnamamide derivatives, several structure-activity relationships were found including an influence of substituents of phenyl ring and olefin linker, a length of the olefin linker, and a structure of the amide moiety [10,11]. Various cinnamamide derivatives showed broad anticonvulsant activity in several models of seizures and epilepsy in mice and/or rats like maximal electroshock (MES), subcutaneous metrazol (*sc*MET), intravenous metrazol seizure threshold (*iv*MET), isoniazid-induced convulsion, 6-Hz psychomotor seizure model, corneal kindled mouse, hippocampal kindled rat, and lamotrigine-resistant amygdala kindled rat models [11,12,13]. Their molecular mechanism of action was also investigated and some molecular targets were recognized including GABA_A_ receptor, serotonergic receptors, vanilloid receptor (TRPV1), and dopamine pathway [11,14,15,16,17]. 

**Figure 1:**
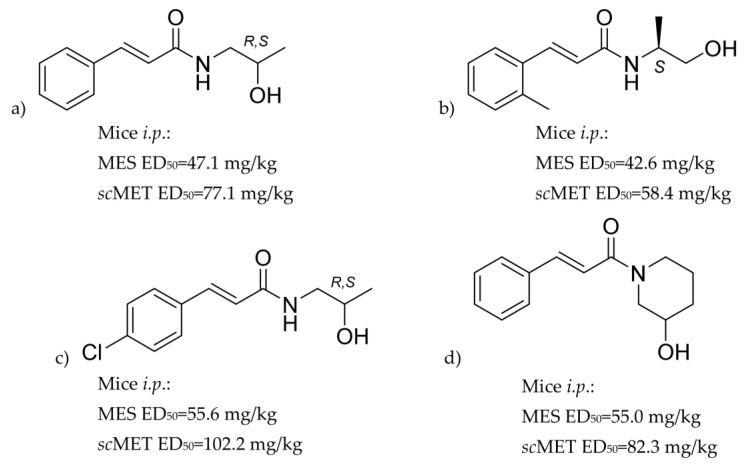
Structures of previously reported cinnamamide derivatives with anticonvulsant activity: (**a**) *R,S*-(2*E*)-*N*-(2-hydroxypropyl)-3-phenylprop-2-enamide [19], (**b**) *S*-(2*E*)-*N*-(1-hydroxypropan-2-yl)-3-(2-methylphenyl)prop-2-enamide [21,22], (**c**) *R,S*-(2*E*)-3-(4-chlorophenyl)-*N*-(2-hydroxypropyl)prop-2-enamide [21,22], (**d**) *R,S*-(2*E*)-1-(3-hydroxypiperidin-1-yl)-3-phenylprop-2-en-1-one [17].

**Figure 2:**
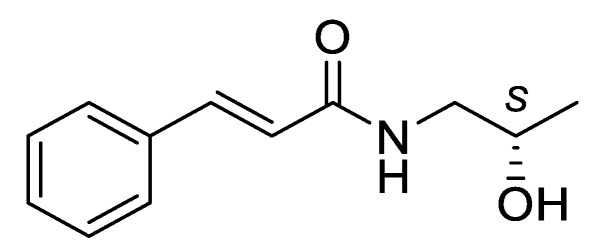
The structure of the title compound: *S*(+)-(2*E*)-*N*-(2-hydroxypropyl)-3-phenylprop-2-enamide, KM-568.

Our former studies concerned several *N*-substituted cinnamamide derivatives with promising anticonvulsant activity in various animal models of seizures and epilepsy [17,18,19,20,21,22]. Most active compounds showed activity in standard tests like MES and *sc*MET but also in a 6-Hz test considered as a model of resistant seizures as well as in kindling models considered as potential predictors of antiepileptogenic activity (Figure 1). Among them, there was *R,S*-*N*-(2-hydroxypropyl)cinnamamide (Figure 1a), unsubstituted in a phenyl ring racemic derivative of 1-aminopropan-2-ol [19]. The beneficial anticonvulsant properties of this compound were a premise for synthesis and evaluation of its pure enantiomers. Herein, we report anticonvulsant activity of its *S* enantiomer (compound KM-568, Figure 2). We used spectroscopy, elemental analysis and crystallography to confirm its chemical structure and configuration. The compound was investigated in several animal models of seizures and epilepsy. Some attempts were also made to identify its mechanism of action. Additionally, we performed preclinical safety assessment of the tested compound including in vitro metabolism studies, mutagenicity, and cytotoxicity assays to ensure appropriate properties required for novel drug candidates.

## 2. Results

### 2.1. Synthesis and Crystal Structure

**Figure 3:**
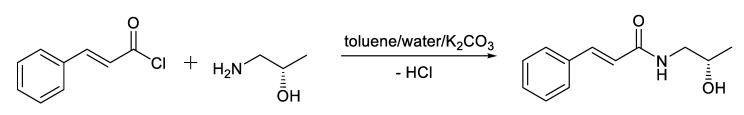
Synthesis of compound KM-568.

The title compound was synthesized in an *N*-acylation reaction using *E*-cinnamoyl chloride and *S*-1-aminopropan-2-ol according to Figure 3. The reaction was carried out in a two-phase solvent system toluene/K_2_CO_3_ solution with a satisfactory yield of 79%. The structure was confirmed by means of spectroscopic methods (^1^H NMR, ^13^C NMR, LC/MS) and elemental analysis while purity was confirmed by means of HPLC. 

**Figure 4:**
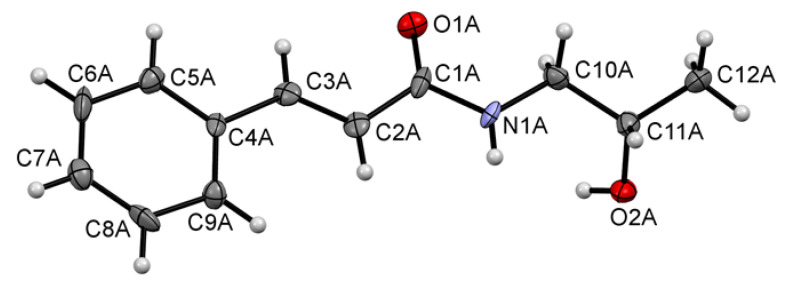
The molecular structure of molecule A showing the atom numbering scheme. Displacement ellipsoids are drawn at the 50% probability level.

In Figure 4, the molecular conformation of the investigated compound in the crystal with the atom numbering scheme for molecule A is shown. This compound crystallizes with eight molecules in the asymmetric unit and the molecules are marked as A, B, C, D, E, F, G and H. The crystal structure confirms the assignment of the E isomer (double bond C2=C3) in all eight molecules. Likewise, all molecules possess *S* configuration at the chiral carbon atom C11. 3-Phenylprop-2-enamide moiety is almost planar with the values of interplanar angle between the plane of aromatic ring and the plane of amide group being 9.9(8), 16.5(7), 16.6(7), 16.3(6), 18.6(7), 11.1(8), 16.9(6) and 15.3(6)°, for molecules A, B, C, D, E, F, G and H, respectively. We have observed similar values earlier in the crystal structures of other cinnamamide derivatives [19,21,23,24]. It is worth notingthat this angle for determined crystal structure of racemic mixture of KM-568 has value 18.7(1)° [23]. 

**Figure 5:**
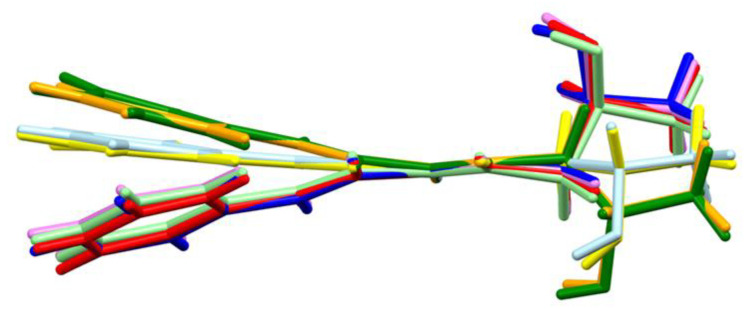
The overlap of amide groups of eight molecules: A yellow, B orange, C red, D light green, E green, F light blue, G blue and H violet.

The eight molecules differ not only in planarity but also in conformation of substituent at nitrogen atom (Figure 5). These differences are visible by comparison of the torsion angle C1-N1-C10-C11 being −169.4, −127.2, 84.3, 87.1, −128.1, −172.1, 79.0 and 79.3° for molecules A, B, C, D, E, F, G and H, respectively. The most favourable conformation, which adopts four molecules, is with the torsion angle in the range from 79 to 87°.

**Figure 6:**
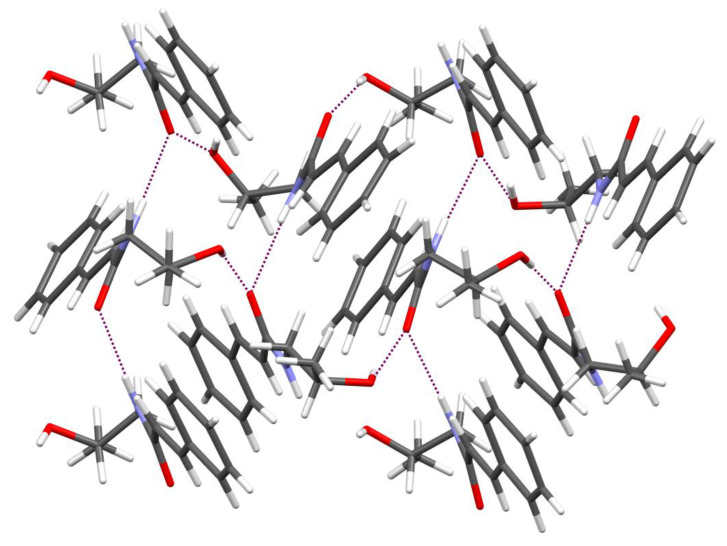
Partial packing view of KM-568 molecules projected along [010] direction. Dashed lines indicate hydrogen bonds.

From a topological point of view, there are two groups (N-H and O-H) as donors of hydrogen atoms and one as an acceptor (C=O), which can generate intermolecular hydrogen bonds. The packing of molecules is determined by N-H···O and O-H···O intermolecular hydrogen bonds (Figure 6). These interactions create the $R_{4}^{2}$(14) and $R_{4}^{4}$(22) [25].

### 2.2. Anticonvulsant Activity

The compound KM-568 was tested for anticonvulsant and analgesic activity within Anticonvulsant Screening Program held by National Institutes of Health (NIH, USA). It was tested in mice and rats in intraperitoneal (*i.p*.) and/or oral (*p.o*.) routes of administration in various models of seizures and one model of hyperalgesia. 

In Table 1 and Table 2, there are presented results obtained for KM-568 in acute seizure models. Table 1 contains the results of tests performed in mice in the maximal electroshock test (MES), 6-Hz psychomotor seizure models (6-Hz), seizures induced by chemoconvulsants: metrazol (*sc*MET), bicuculine and picrotoxin, and neurotoxicity evaluation (TOX). MES, *sc*MET, and TOX evaluations were performed after intraperitoneal and oral administration of compound, while there were other tests after intraperitoneal administration. In Table 2, results are presented of tests performed in rats in MES, *sc*MET as well as in neurotoxicity evaluation (TOX) after intraperitoneal and oral administration. 

KM-568 showed beneficial anticonvulsant activity in both electrically and chemically induced seizure models. Thus, the results suggest that it is able to inhibit seizure spread (verified in MES), raise the chemoconvulsant-induced seizure threshold of an animal (verified in tests involving metrazol and picrotoxin) as well as block psychomotor seizures (verified in 6-Hz tests). Moreover, 6 Hz models in mice involving 32 mA and 44 mA currents are considered as models of drug resistant epilepsy, so the activity of compound KM-568 is especially beneficial. The dose–response relationship was observed in all tests (except of bicuculine assay) and appropriate ED_50_s and TD_50_s were obtained (reported in Table 1 and Table 2).

Compound KM-568 was further tested in mice. In genetically modified Frings audiogenic seizure-susceptible mice, it showed high efficacy with ED_50_ of 13.21 mg/kg (*i.p*.) (Table 3). The proved properties provided beneficial information about anticonvulsant potential and brain bioavailability following systemic administration. In an intravenous metrazol seizure threshold test (*iv*MET), compound KM-568 increased the time causing first twitch and first clonus during metrazol infusion in a dose dependent manner so it actually raised a seizure threshold at which a seizure can be induced by metrazol and proved anticonvulsant potential (Table 4). The *iv*MET test enables identification of molecules that affects GABAergic neurotransmission [26] so the proved in vivo activity of compound KM-568 may suggest its molecular mechanism of action.

Compound KM-568 was tested in mice in a mesial temporal lobe epilepsy model. This model is considered as a model of temporal lobe epilepsy in humans and classified as a chronic seizure model. The tested compound at the dose of 114 mg/kg (*i.p*.) decreased the number of digital EEG recorded hippocampal paroxysmal discharges (HPD) to the level of 73.9% of the baseline showing moderate activity (Table 5). Another chronic seizure model, the corneal kindled mouse model, provided more satisfactory results for compound KM-568. The compound protected tested animals from developing seizures at the doses in the range of 57–115 mg/kg (0.25 h after *i.p*. administration), which resulted in obtaining the ED_50_ of 79.17 mg/kg (Table 6). This activity proved not only anticonvulsant but also antiepileptogenic potential of compound KM-568.

Further tests were also performed in rats. In a hippocampal kindled rat model, the tested compound significantly decreased seizure score (0.25 h after *i.p.* injection at the dose of 27 mg/kg) and seizure duration (0.75 h and 2.25 h after *i.p*. injection at the dose of 27 mg/kg) (Table 7). This animal test is considered as a model of secondary generalized focal seizures in humans and is similar to other kindling models, which may be classified as a model of epileptogenesis [27]. Moreover, in a lamotrigine (LTG) resistant amygdala kindled rat model, compound KM-568 significantly reduced the seizure score at a dose of 80 mg/kg (0.25 h after *i.p.* administration) (Table 8). This model was proposed as a model of drug refractory epilepsy because LTG-resistant kindled rats are drug-resistant to voltage-gated sodium channel blockers: lamotrigine, carbamazepine, phenytoin, and voltage-gated calcium and sodium channels blocker: topiramate, remaining susceptible to GABAergic transmission modulator: valproate, GABAergic transmission modulator and excitatory amino acids receptors blocker: felbamate, as well as voltage-gated potassium channel blocker: retigabine [28,29].

Compound KM-568 was tested in rats at the doses in the range 200–400 mg/kg *i.p.* in a pilocarpine-induced status epilepticus model. After administration of pilocarpine, at the time point when a convulsive effect of at least level 3 in the Racine scale was noted (time 0 h) and 0.5 h afterwards (time 0.5 h), KM-568 was administered intraperitoneally at a certain dose. Significant protection was observed at both time-points, for time 0.5 h, a dose–effect relationship was additionally studied and resulted in ED_50_ of 297.45 mg/kg (*i.p*.) and ED_97_ of 498.2 (378.3–1526.9) mg/kg (*i.p*.) (Table 9).

### 2.3. Test of Hyperalgesia

Compound KM-568 was tested in a mice test of hyperalgesia employing formalin. Compound KM-568 was administered at the dose of 44 mg/kg *i.p*., corresponding with an MES ED_50_ dose, giving results reported in Table 10. KM-568 reduced the time of licking of the affected paw by 24% in an acute phase and 32% in an inflammatory phase, but the results were not statistically significant.

### 2.4. Molecular Mechanism of Action

In order to identify molecular mechanism of action, KM-568 was tested at 10 µM in three radioligand binding assays: rat cerebral cortex GABA receptor non-selective agonist radioligand, AMPA (α-amino-3-hydroxy-5-methyl-4-isoxazolepropionic acid) receptor agonist radioligand, and NMDA (*N*-methyl-D-aspartate) receptor antagonist radioligand as well as one functional assay involving a human recombinant TRPV1 receptor expressed in CHO cells (agonist and antagonist effects). No significant effects were observed at any of the tested targets.

### 2.5. Preclinical Safety Evaluation

In in vitro metabolism simulation performed by means of a mouse liver microsome model, compound KM-568 demonstrated half-life time (t_1/2_) of 128.33 min and internal clearance (Cl_int_) value of 13.5 µL/mg min. After 60 min of incubation with microsomes in test conditions, 72.37% of the compound remained unchanged. The obtained results allow for qualifying it as a moderate metabolically stable entity. Compound KM-568 was more susceptible to in vitro metabolism when compared to its *p*-chloro substituted analog [21], but significantly more stable than other reported cinnamamides [30]. In the study, specific metabolites were not identified, which may be associated with metabolism to low molecular weight molecules.

The results obtained in mutagenicity evaluation showed that there were no doses of KM-568 with more than a 2-fold induction of the number of positive wells (number of revertants) over the solvent control baseline, and a dose dependent response was not observed (Table 11). Therefore, KM-568 did not exhibit mutagenic activity in the assay. 

**Figure 7:**
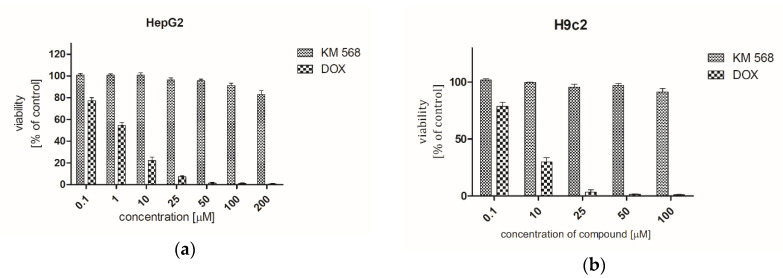
Results of cytotoxicity evaluation of compound KM-568 and doxorubicin (DOX) in (**a**) HepG2 and (**b**) H9c2 cell lines in an MTT assay. Results were presented as mean ± SD from three samples.

**Figure 8:**
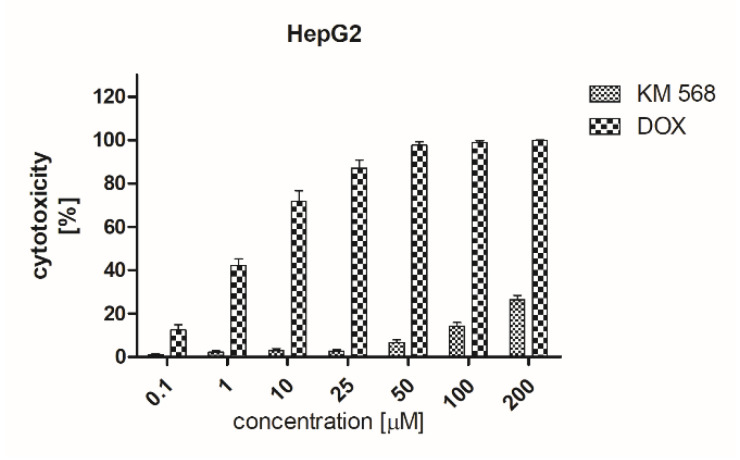
Results of cytotoxicity evaluation of compound KM-568 and doxorubicin (DOX) in an HepG2 cell line in an LDH assay. Results presented as mean ± SD from three samples.

A cytotoxic effect of compound KM-568 was evaluated in two cell lines: human liver cancer cells (HepG2) and rat cardiomyoblasts (H9c2) by means of an MTT colorimetric assay which detects the content of viable cells in the sample on the basis of reduction of yellow MTT to its purple formazan derivative. In HepG2 cells, an additional cytotoxicity assay based on the activity of lactate dehydrogenase of living cells was performed. The results obtained in these experiments are presented in Figure 7 and Figure 8. They indicated that KM-568 had no cytotoxic effect up to 100 µM in HepG2 and H9c2 cells. The percent of living cells after 24 h incubation with 100 µM KM-568 was close to 100%.

## 3. Discussion

Searching for new anticonvulsant and antiepileptic agents constitutes an important issue within modern medicinal chemistry. Despite availability of about 30 antiseizure drugs on the market, it is estimated that about 30% of seizures still remain resistant to pharmacotherapy [31]. Cinnamic acid derivatives, including those possessing amide moiety i.e., cinnamamide derivatives, have been recognized as important drug candidates for various neurological disorders including epilepsy [10,11]. Two significant examples of cinnamamide derivatives are antiepilepsirine and cinromide. Antiepilepsirine, primarily synthesized as a derivative of naturally occurring compound—piperine, was marketed as an antiepileptic drug [32], while cinromide entered clinical trials for the treatment of the Lennox–Gastaut syndrome [33]. Several other cinnamamide derivatives were tested in animal models of seizures and have been reported for several decades as potential drug candidates for epilepsy [10,11].

Animal models were designed to mimic specific symptoms and disorders in humans. Two animal models, maximal electroshock and subcutaneous metrazol tests, were considered for decades as ‘gold standards’ in searching for novel antiseizure drugs and they enabled identification of compounds effective in humans in generalized tonic-clonic seizures and generalized non-convulsive seizure, respectively. Since the utilization of those two models did not provide drug candidates for refractory symptoms, several other animal models were introduced to preclinical programs aiming to search for a new treatment for epilepsy [31,34]. For example, very important models are those involving kindling procedures, such as corneal-kindled mouse or hippocampal kindled rat models, because they are considered as epileptogenesis models as well as predictors of clinical efficacy for complex partial seizure in humans. Thus, compounds active in kindling models could probably inhibit the development of the disease [35]. 

*S*(+)-*N*-(2-hydroxypropyl)cinnamamide (compound KM-568) presented here showed anticonvulsant activity in several models of epilepsy and seizures in mice and rats. It was active in a genetic animal model of epilepsy (Frings audiogenic seizure-susceptible mouse model), acute seizures induced electrically (maximal electroshock test, 6-Hz psychomotor seizure model), chronic seizures induced electrically (corneal kindled mouse model, hippocampal kindled rat model, lamotrigine-resistant amygdala kindled seizure model in rats), acute seizures induced chemically (subcutaneous metrazol seizure threshold test, seizures induced by picrotoxin in mice) and in the status epilepticus model with spontaneous recurrent seizures induced by pilocarpine. Compound KM-568 was presented here and reported its racemic form previously. *R*,*S*-*N*-(2-hydroxypropyl)cinnamamide (Figure 1a) showed comparable values of pharmacological parameters in preliminary evaluation (mice, *i.p*.: MES 47.1 and 44.46 mg/kg, *sc*MET 77.1 and 104.29 mg/kg, rats *p.o*.: MES 22.8 and 30.81 mg/kg, respectively) [18]. Enantiomer *R* showed slightly less promising preliminary results and pharmacological parameters were not established for it. In case of chiral compounds, racemate as well as both enantiomers should be tested for activity and safety. Finally, only compound KM-568, enantiomer *S*, was chosen for more advanced examination and racemate as well as *R* enantiomer were characterized only in preliminary assays.

Utilization of animal models is widely accepted in antiseizure and antiepileptic drugs development programs. They enable identification of an active derivative regardless its molecular mechanism of action as well as acting through more than one molecular targets [33]. Cinnamamide derivatives tested for anticonvulsant activity were suspected to influence GABAergic transmission because of their in vivo activity profile [36]. In vitro tests proved various affinities for the GABA_A_ receptor, regarding structural modifications of the tested cinnamamide derivatives with best results proved for compounds possessing two olefin linkers (four carbon atoms) between phenyl ring and amide group [14,15,16]. *N*-methyl-D-aspartate (NMDA) receptor was identified as a molecular target for selected *N*-(phenylalkyl)cinnamamides [37], vanilloid receptor (TRPV1) for selected *N*-arylcinnamamides [38], while serotonergic and/or dopamine pathways for selected aminoalkanol derivatives of cinnamic acid, similar to compound KM-568 [17,21].

Works reported here aiming for identification of a molecular mechanism of action of the compound KM-568 were not satisfactory. Tests regarding its affinity for GABA and NMDA receptors as well as functional assay in a TRPV1 receptor did not provide evidence for influencing those molecular targets. However, some assumptions may be made on the basis of in vivo activity of KM-568. The proved anticonvulsant activity in LTG-resistant amygdala kindled rats suggests that the compound does not act through modulation of voltage-dependent sodium channels [34]. On the other hand, the ability of the compound to prevent the occurrence of seizures after administration of a non-competitive GABA_A_ receptor blocker, and picrotoxin may indicate involvement of GABAergic transmission in its molecular mechanism of action [39,40]. Similarly, effectiveness in models of seizures induced by metrazol, which is likely to produce its convulsive effect via the GABA receptor complex [41], may indicate the GABA system as a molecular mechanism of action. Metrazol was also proved to increase calcium and sodium influx in neurons as well as to cause the calcium channels to lose their selectivity and conduct sodium ions. Further studies proved that L-type calcium channel ligands were able to antagonize convulsive effect of metrazol [42] so this could be another molecular mechanism of action of compound KM-568. 

The performed preclinical safety assessment of the tested compound included in vitro metabolism studies, mutagenicity evaluation, and cytotoxicity in two cell lines (HepG2 and H9c2) assays. In vitro determination of metabolic profile of new chemical entities is an important step within the drug discovery process, since it influences pharmacokinetic characteristics of therapeutic compounds [43]. Our in vitro studies showed that KM-568 is a moderately stable molecule. In vivo studies showed that its time-to-peak effect for anticonvulsant activity in mice was 0.25 h and 0.25–0.5 h for intraperitoneal and oral administration, respectively. In rats, time-to-peak effect was 0.25 h for intraperitoneal and 1.0 h for oral administration. Previously reported cinnamamide derivatives possessed similar properties in vivo [19,21]. Mutagenicity evaluation has become an essential component in the preclinical safety assessment of drug candidates and compound KM-568 showed no mutagenic properties in a microplate Ames assay. In the absorptive phase, the liver is exposed to high concentrations of xenobiotics, and it is also the primary organ responsible for metabolism of drugs. In the deceased, liver toxicity was identified as a relatively rare but serious adverse effect of currently used antiseizure drugs such as valproic acid, carbamazepine, lamotrigine, levetiracetam, and ethosuximide [7,44]. Hepatocytotoxicity assessment in HepG2 cells showed that KM-568 is safe in concentrations up to 100 µM.

## 4. Materials and Methods 

The CAS number of the compound KM-568 is 1609486-25-0. It was a subject of our patent claim [45].

### 4.1. Chemistry

Reagents (*E*-cinnamoyl chloride, *S*-2-aminopropan-1-ol) were purchased from Alfa Aesar (Kandel, Germany). Solvents were commercially available materials of reagent grade. Melting point (m.p.) was determined using a Büchi SMP-20 apparatus (Büchi Labortechnik, Flawil, Switzerland) in an open capillary. The ^1^H NMR spectrum was recorded in CDCl_3_ by means of a Varian Mercury-VX 300 NMR spectrometer (Varian Inc., Palo Alto, CA, USA). The ^13^C NMR spectrum was recorded in CDCl_3_ by means of JOEL 500MHz spectrometer (Joel Ltd., Tokyo, Japan). The results of ^1^H NMR are presented in the following format: chemical shift δ in ppm, multiplicity, coupling constant *J* in Hertz (Hz), number of protons, protons’ position. Multiplicities are showed as the abbreviations: s (singlet), bs (broad singlet), d (doublet), m (multiplet). Results of ^13^C NMR are presented in the following format: chemical shift δ in ppm, ^13^C’s position; all signals were singlets. Elemental analysis was performed on a Vario EI III elemental analyzer (Elementar Analysensysteme GmbH, Langenselbold, Germany). The IR spectrum was recorded on a Jasco FT/IR 410 spectrometer (JASCO Deutschland GmbH, Pfungstadt, Germany) using KBr pellets and are reported in cm^−1^. The mass spectrum was obtained on a Waters ACQUITYTM TQD system with the TQ detector (Waters, USA). The ACQUITY UPLC BEH C18 50 mm column was used (Waters Corporation, Milford, MA, USA). The thin-layer chromatography (TLC) was carried out on pre-coated aluminum sheets (silica gel 60 F-254, Merck, Darmstadt, Germany). Spots were visualized by UV light. Specific rotation was measured on a Jasco P-2000 polarimeter (JASCO Deutschland GmbH, Pfungstadt, Germany).

#### 4.1.1. General Procedure of Preparation of Compound KM-568

Synthesis of the tested compound KM-568 was carried out according to scheme presented in Figure 3. In the reaction, 0.015 mole of *S*-2-aminopropan-1-ol was dissolved in 50 mL 5.5% solution of K_2_CO_3_. Then, 10 mL toluene was added and the solution of 0.015 mole of *E*-cinnamoyl chloride in 30 mL of toluene was added dropwise while the reaction mixture was mixed on magnetic stirrer. The crude product obtained in the reaction was filtered and washed with 20 mL of 5% K_2_CO_3_ and 50 mL of distilled water. The compound was dried and crystallized with a mixture of *n*-hexane and toluene (3:1 *v/v*).

#### 4.1.2. Analytical Data for Compound KM-568 - S(+)-(2E)-N-(2-hydroxypropyl)-3-phenylprop-2-enamide

White solid, m.p. 121–123°C. M=205.25. Anal. C_12_H_15_NO_2_ calcd: C, 70.22; H, 7.37; N, 6.82. Found: C, 70.06; H, 7.47; N, 6.80. ^1^H-NMR (CDCl_3_, 300 MHz) 1.24 (d, *J* = 3.4; 3H, CH_3_); 2.64 (d, *J* = 4.4; 1H, OH); 3.21–3.30 (m, 1H, NH-CHH); 3.56–3.63 (m, 1H, NH-CHH); 3.97–4.04 (m, 1H, CH(OH)-CH_3_); 6.12 (bs, 1H, NH); 6.43 (d, *J* = 15.6; 1H, Ar-CH=CH); 7.34–7.41 (m, 3H, Ar-H); 7.47–7.52 (m, 2H, Ar-H); 7.64 (d, *J* = 15.6; 1H, Ar-CH=CH). ^13^C NMR (CDCl_3_, 126 MHz) 21.07 (-CH_3_), 47.42 (-CH_2_-), 67.57 (-CH-), 120.39 (=CH-), 127.92 (Ar-C2, Ar-C6), 128.92 (Ar-C3, Ar-C5), 129.89 (Ar-C4), 134.72 (Ar-C1), 141.56 (-CH=), 167.16 (C=O). IR (cm^−1^): 3304; 3066; 2971; 1653; 1607; 1561. ESI-MS: [M+H]^+^ calcd for C_12_H_16_NO_2_ = 206.11, [M+H]^+^ found = 206.18. R_f_=0.72 (methanol:ethyl acetate 1:1); 0.63 (chloroform:methanol 9:1), 0.75 (chloroform:methanol 5:1); [*α*]_D_^20^ = 22.77 deg dm^−1^cm^3^g^−1^ (c = 0.02 gcm^−3^ in methanol); miLogP = 1.77.

### 4.2. Crystallographic Data for KM-568

Single crystals suitable for an X-ray structure analysis were obtained from butyl acetate by slow evaporation of the solvent at room temperature. 

Intensity data were collected using the XtaLAB Synergy-S diffractometer (Rigaku Oxford Diffraction, Wrocław, Poland), equipped with the Cu (1.54184 Å) Kα radiation source and graphite monochromator. The phase problem was solved by direct methods using SIR-214 [46] and all non-hydrogen atoms were refined anisotropically using weighted full-matrix least-squares on F^2^. Refinement and further calculations were carried out using SHELXL-2014 [47]. The hydrogen atoms bonded to carbon atoms were included in the structure at idealized positions and were refined using a riding model with U_iso_(H) fixed at 1.2 U_eq_ of C and 1.5 U_eq_ for methyl groups. 

Hydrogen atoms attached to nitrogen and oxygen atoms were found from the difference Fourier map and refined without any restraints. For molecular graphics, the MERCURY [48] program was used.

KM-568: C_12_H_15_NO_2_, M_r_ = 205.25, crystal size = 0.14 × 0.26 x 0.33 mm^3^, monoclinic, space group P2_1_, a = 9.3021(1) Å, b = 28.9457(2) Å, c = 16.2741(1) Å, V = 4381.89(6) Å^3^, Z = 16, T = 100(2) K, 141,765 reflections collected, 18,060 unique reflections (R_int_ = 0.0577), R1 = 0.1005, wR2 = 0.2282 [I > 2σ(I)], Flack parameter 0.06(5).

CCDC 2,004,943 contains the supplementary crystallographic data. These data can be obtained free of charge from The Cambridge Crystallographic Data Centre via www.ccdc.cam.ac.uk/data_request/cif.

### 4.3. In Vivo Pharmacology—General

In vivo activity testing was performed at the Epilepsy Branch, National Institute of Neurological Disorders and Stroke, National Institutes of Health (NIH, Bethesda, USA) within the Anticonvulsant Screening Program (ASP) (currently known as the Epilepsy Therapy Screening Program (ETSP)). Pharmacological evaluation was planned and performed in accordance with the Institute of Laboratory Resources polices on the humane care of laboratory animals and approved by the University of Utah’s Institutional Animal Care and Use Committee (IACUC). All testing protocols were previously published [49,50] as well as they are available on the NIH website [51]. In the tests, albino mice Carworth Farms No.1 (CF-1) (Charles River), C57/Bl6 mice (Charles River), Frings mice (University of Utah) or male rats Sprague–Dawley (Charles River) were used. All animals were housed, fed, and handled in a manner consistent with the recommendations in the National Council Publication, ‘Guide for the Care and Use of Laboratory animals’ [51]. The tested compound was suspended in 0.5% methylcellulose.

### 4.4. In Vivo Pharmacology Procedures

#### 4.4.1. Maximal Electroshock Test (MES)

After intraperitoneal or oral administration of compound KM-568 at certain dose, an alternating current at 60 Hz and 50 mA (mice) or 150 mA (rats) was delivered for 0.2 sec via corneal electrodes to induce seizures. Prior to each stimulation, a drop of 0.5% tetracaine hydrochloride in saline was applied to cornea. Protection was defined as the abolition of the hindlimb tonic extension of the seizures. ED_50_s and 95% confidence intervals were determined by probit analysis [49,50,51].

#### 4.4.2. Subcutaneous Metrazol Seizure Threshold Test (scMET)

The tested compound was administered *i.p*. or *p.o*. at certain dose. Then, subcutaneous injection of metrazol (*syn*. pentetrazol, pentylenepentetrazol) dissolved in 0.9% NaCl at the dose of 85 mg/kg (mice) or 70 mg/kg (rats) was performed to induce seizures. Protection was scored if there was not an observed episode of clonic spasm of at least 5 sec. ED_50_s, and 95% confidence intervals were determined by probit analysis [49,50,51].

#### 4.4.3. Neurotoxicity (TOX)

In mice, the neurological deficit was measured by the rotarod test in which a 1 inch diameter knurled plastic rod rotating at 6 rpm was used. Tests were performed at 0.25 h or 0.5 h of intraperitoneal or oral administration of the certain dose of the compounds KM-568. Each mouse was placed on the rod. If the animal was not able to remain on the rod for at least 1 min in each of three trials, the neurotoxicity was scored. In rats, the neurological deficit was measured in behavioral tests and scored when ataxia and loss of placing response and muscle tone were observed. Tests in rats were performed after 0.5 h of intraperitoneal administration or 1.0 h of oral administration of the certain dose of compound KM-568. TD_50_s were calculated by probit analysis [49,50,51].

#### 4.4.4. 6-Hz Psychomotor Seizure Model (6-Hz)

The test was performed after 0.25 h (for 32 mA current) or 0.5 h (for 44 mA current) of *i.p*. administration of compound KM-568. The groups of 8 animals were challenged for a single dose of the tested compounds. In addition, 0.2 ms pulses of electric current at 6 Hz and 32 mA or 44 mA were delivered for 3 sec via corneal electrodes. The mouse was released into an observation cage immediately after stimulation. The electric stimulation in a 6-Hz test causes a minimal clonic phase followed by stereotyped, automatic behaviors. The seizure activity can be described as stun, forelimb clonus, twitching of the vibrissae, and Straub tail. The protection was scored if the mouse resumed its normal activity within 10 sec from the stimulation. ED_50_s and 95% confidence intervals were determined by probit analysis [49,50,51].

#### 4.4.5. Chemoconvulsants Tests—Bicuculine and Picrotoxin

Initially, compound KM-568 was administered *i.p*. at 130.0 mg/kg in two groups of CF-1 mice (*n* = 8). After 0.25 h, the subcutaneous injections of either bicuculine (2.7 mg/kg) or picrotoxin (2.5 mg/kg) were performed. The, animals were placed in isolation cages and observed for 30 min (in a bicuculine assay) or 45 min (in a picrotoxin assay) for the presence or absence of an episode of a clonic seizure. Compound KM-568 was further tested in lower doses in picrotoxin assay; then, ED_50_ and 95% confidence interval were determined by probit analysis [51].

#### 4.4.6. The Frings Audiogenic Seizure-Susceptible Mouse Model (AGS-Susceptible Mouse Model)

Frings audiogenic seizure-susceptible mice are genetically susceptible to sound-induced reflex seizures. Mice (18–25 g) were administered *i.p.* compound KM-568 at the doses in the range of 10.0–17.5 mg/kg. Fifteen minutes later, an individual mouse was placed in a round plexiglass jar (15 cm diameter, 18 cm high) and exposed to a sound stimulus of 110 decibels (11 kHz) delivered for 20 sec. Mice were observed for 25 sec for the presence or absence of hind limb tonic extension. Protection was scored if the mouse did not display hind limb tonic extension. ED_50_ and the 95% confidence interval were calculated by probit analysis [51,52].

#### 4.4.7. Intravenous Metrazol Seizure Threshold Test (ivMET)

Male CF-1 mice (n = 10 per dose) were injected intraperitoneally the vehicle, MES ED_50_ (44 mg/kg) or TD_50_ (148 mg/kg) of KM-568. Then, 0.5% metrazol solution was then infused at a previously determined time-to-peak effect (0.25 h). The infusion was done at a constant rate of 0.34 mL/min through a tube cannulating a lateral tail vein of a test animal. The time from the start of the infusion to the appearance of the first twitch (time to twitch in sec) and the onset of sustained clonus (time to clonus in sec) were recorded. These times were also converted to the dose of metrazol (in mg/kg) causing first twitch and clonus [26].

#### 4.4.8. Mesial Temporal Lobe Epilepsy Model (MTLE) in Mice

Adult male C57/Bl6 mice were placed in a stereotactic frame, injected with kainate (1 nmol/100 nL) and implanted with 1 bipolar electrode in the dorsal hippocampus. The animals were then allowed to recover for four weeks. Four animals chosen for tests were intraperitoneally injected with KM-568 at 114 mg/kg (ED_50_ obtained in 6-Hz 44 mA test). Digital EEG recordings were performed on freely moving animals for 20 min pre-injection (reference period) and from 20 to 40 min after injection (10 min before and 10 min after peak time of effect in a 6-Hz 44 mA test). Animals are used as their own controls. In the testing periods, spontaneous recurrent hippocampal paroxysmal discharges (HPD) were counted [51].

#### 4.4.9. Corneal Kindled Seizure Model in Mice

Mice were stimulated via corneal electrodes (3 mA, 60 Hz, 3 sec) for an average of 12 days, 4 h apart. Prior to each stimulation, a drop of 0.5% tetracaine hydrochloride in saline was applied to the cornea. After development of five consecutive seizure stages at level 5 according to the Racine scale, animals were considered kindled. The groups of 8 mice received an intraperitoneal injection of appropriate doses of KM-568. At previously found time-to-peak effect (0.25 h), the animals were challenged with the corneal kindling stimulus (3 mA, 3 sec). Mice were scored as protected if the seizure score was less than or equal to three and not protected if the seizure score was more than three, based on the Racine scoring system [51,53,54].

#### 4.4.10. Hippocampal Kindled Rat Model

The electrodes were implanted in brains one week prior to experiments. Then, rats were stimulated with suprathreshold stimuli of 200 µA for 10 sec at 50 Hz every 30 min for 6 h on alternate days until fully kindled. One week after reaching the fully kindled state, the effect of 23 and 27 mg/kg, *i.p*. of KM-568 in the groups of 5–6 rats were evaluated. The tests were performed at different time points (0–2.25 h) after compound administration. The results were scored as behavioral seizure score (according to Racine scale) and after discharge duration. The protection was noted if the seizure score was 3 or less [27,51].

#### 4.4.11. Lamotrigine (LTG) Resistant Amygdala Kindled Seizure Model in Rats

Anesthetized male Sprague–Dawley rats (250–300 g) were surgically implanted with an electrode into the left amygdala [49]. After a one-week recovery period, kindling was initiated. A suprathreshold stimulus (200 µA) was delivered daily until all animals display stage 4–5 seizures. Lamotrigine at 5 mg/kg *i.p*. was also administered daily during kindling acquisition. One week after all animals were kindled, the animals received a challenge dose of lamotrigine (30 mg/kg, *i.p*.) to confirm LTG-resistance. On the 4th day, compound KM-568 was administered *i.p*. at the doses 20, 40, and 80 mg/kg. Fifteen minutes after administration, rats were challenged with the kindling stimulus. Results were provided as seizure score (according to Racine scale, protection defined as less than 3) and after discharge duration (in sec) [51].

#### 4.4.12. Pilocarpine-Induced Status Epilepticus Model

Pilocarpine was administered intraperitoneally into rats at the dose of 50 mg/kg. Each animal was being observed. The time point when the convulsive effect at the level of at least 3 in the Racine scale was noted (time 0 h), and, 0.5 h (time 0.5 h) afterwards, KM-568 was administered. The protection was scored if the administration of the compound inhibited further seizure activity [53,55,56].

#### 4.4.13. Formalin Test of Hyperalgesia

0.05% formalin was injected subdermal to the hindpaws of mice, which caused licking. In the tested group (*n* = 8), KM-568 was administered intraperitoneally 0.25 h prior to formalin at the dose of 44 mg/kg. In the control group, methylcellulose was used. Animals were then observed for the first 2 min of each 5 min for a total of 40 min. The cumulative duration of licking (in sec) during each 2-min recording period was measured for analysis across compound and vehicle-pretreated groups [57].

### 4.5. Molecular Mechanism of Action

Tests were performed by Eurofins Cerep (Eurofins CEREP SA, Celle-Lévescault, France) as a commercial service. In radioligand assays, rat cerebral cortex GABA receptor as well as rat cerebral cortex AMPA (α-amino-3-hydroxy-5-methyl-4-isoxazolepropionic acid) and NMDA (*N*-methyl-D-aspartate) receptors with 10 nM [^3^H]GABA, 8 nM [^3^H]AMPA, and 5 nM [^3^H]CGP39653, respectively, were used. The results were detected by means of scintillation counting. In functional assays, a human recombinant TRPV1 receptor expressed in Chinese hamster ovary (CHO) cells with 1 µM capsaicin or 30 nM capsazepine were used. Fluorimetry was used as a detection method. 

### 4.6. In Vitro Metabolism

Stock solution of KM-568 was prepared in methanol. Mouse liver microsomes (Sigma–Aldrich, Darmstadt, Germany) were carefully thawed on ice before the experiment. A compound tested at concentration 20 μM was preincubated with mouse liver microsomes (0.4 mg/mL) in 100 mM potassium-phosphate buffer (pH 7.4) for 15 min at 37 °C. The biotransformation reaction was started with the addition of NADPH-regenerating system (NADP^+^, glucose-6-phosphate, 0.7 U/mL glucose-6-phosphate dehydrogenase in 100 mM potassium-phosphate buffer) (Sigma–Aldrich, Darmstadt, Germany) and the mixture was incubated at 37 °C. Experiments were carried out 30 and 60 min at 37 °C, in duplicates. Negative control without NADPH-regenerating system was also conducted. Biotransformation was stopped by adding cooled 70% perchloric acid. Next, an internal standard (pentoxifylline) was added and samples were centrifuged at 9000 rpm for 10 min at 4 °C. The supernatant was analyzed by means of LC/MS. In vitro half time (t_1/2_) was established from the slope of the compound depletion curve. Intrinsic clearance (Cl_int_) was obtained from the equation Cl_int_ = (volume of incubation [µl]/protein in the incubation [mg]) × 0.693/t_1/2_ [58,59].

### 4.7. Mutagenicity Assessment

The Ames microplate format (MPF) mutagenicity assay (Xenometrix, Allschwil, Switzerland) was used to test KM-568 mutagenicity. Growth, exposure, and indicator media, positive control chemicals, and four Salmonella typhimurium strains TA98, TA100, TA1535 and TA1537 were included in the kit. TA100 and TA1535 strains detect base substitution mutations, whereas TA98 and TA1537 indicate frameshift mutations. The strains of bacteria included in a kit are in compliance with the Organization for Economic Cooperation and Development (OECD) 471 Guideline for Testing of Chemicals [60].

The assay is a liquid microplate modification of the traditional Salmonella test [61,62]. During the procedure, bacteria are exposed to different concentrations of a test compound for 90 min in a medium containing sufficient histidine to support cell divisions. Then, the cultures are diluted in a pH indicator medium lacking histidine and aliquoted into 48 wells of a 384-well plate. Within 2 days, bacteria that have undergone reversion to amino acid prototrophy will form colonies. Products of bacteria metabolism reduce pH of the medium, changing the color of wells from purple to yellow [63].

The test procedure provided by Xenometrix [63] and described in detail elsewhere [64,65,66] was followed. Bacterial strains were grown overnight (14–16 h) in exposure medium in a shaker incubator and then exposed to KM-568 in 24-well plates for 90 min at 37 °C with agitation. Final KM-568 concentrations in the assay were 0.1, 0.2, and 0.5 mM. After preincubation, the cultures were diluted in the indicator medium and the contents of each 24-well culture were transferred to 48 wells on a 384-well plate and incubated further for 48 h at 37 °C without agitation. After the exposure, the number of wells containing bacteria was scored for yellow wells. Positive controls used for the protocol were 2-nitrofluorene (2-NF) at 2 µg/mL (TA98); 4-nitroquinoline-*N*-oxide (4-NQO) at 0.1 µg/mL (TA100); N4-aminocytidine (N4-ACT) at 100 µg/mL (TA1535) and 9-aminoacridine (9- AAc) at 15 µg/mL (TA1537). Pure DMSO was used as a negative control. All doses were performed in triplicate. 

To evaluate mutagenicity assay results, two criteria were applied: the fold increase in the number of positive wells over the solvent control baseline (FIB), and the dose dependency. The fold increase of revertants relative to the solvent control was determined by dividing the mean number of positive wells at each dose by the solvent control at baseline. The solvent control at baseline was calculated as the mean number of positive wells in the solvent control plus one standard deviation (SD). When an increase of more than twofold relative to the baseline at more than one dose with a dose–response was observed, the sample was classified as positive, whereas when no response > 2 times the baseline and no dose–response was stated, the sample was classified as negative [62,63,64].

### 4.8. Cytotoxicity Assessment

#### 4.8.1. Cell Culture 

Human liver cancer HepG2 cell line (ATCC HB8065) and rat cardiomyoblast cell line H9c2 (ATCC-CRL1446) were purchased in ATCC (American Type Culture Collection, Manassas, VA, USA). The cells were cultured in standard conditions (37 °C, 5% CO_2_) in EMEM medium (ATCC, Manassas, VA, USA), supplemented with 10% FBS (Gibco, Waltham, MA, USA) and antibiotics (1% streptomycin/penicillin mixture, Sigma–Aldrich, Darmstadt, Germany) or DMEM high glucose (ATCC, Manassas, VA, USA) supplemented with 10% FBS (Gibco, Waltham, MA, USA) and antibiotics (1% streptomycin/penicillin mixture, Sigma–Aldrich, Darmstadt, Germany), respectively.

#### 4.8.2. 3-(4,5-dimethylthiazol-2-yl)-2,5-diphenyltetrazolium Bromide (MTT) Assay

The MTT colorimetric assay was used to determine the cytotoxic effects of the KM-568 and DOX (Sigma–Aldrich, Darmstadt, Germany). Cells were seeded at a density of 1 × 10^4^ in 96-well plates. Following overnight culture, the cells were then treated with KM-568 and DOX in concentration range 0.1–200 µM (hepatocytotoxicity) or 0.1–100 µM (cardiocytotoxicity). Following cell exposure to each drug for 24 h in 96 well plates, 10 µL MTT reagent (Sigma–Aldrich, Darmstadt, Germany) was added to each well; after 4 h of incubation (37 °C, 5% CO_2_), the medium was aspirated and formazan produced in cells appeared as dark crystals in the bottom of the wells. Next, 100 µL DMSO was added to each well. Then, absorbance was measured at 570 nm on a multi-well plate reader (Spectra Max iD3, Molecular Devices, San Jose, CA, USA). Each individual experiment was repeated at least three times.

#### 4.8.3. Lactate Dehydrogenase (LDH) Assay

Cells were seeded at density 5 × 10^4^ cells/per well. After 24 h, KM-568 and reference standard DOX were added to final concentrations of 0.1; 0.5; 1.0; 10; 25; 50; 100; 200 µM. After 24 h incubation, plates were centrifuged (400 g, 2 min) and 50 μL of the supernatant was transferred into the corresponding 96-well plate. Subsequently, 50 μL of LDH-reaction mixture was prepared according to the manufacturer’s instructions (Invitrogen, Waltham, MA, USA) and was then added to each well. Incubation was conducted in darkness for 30 min at room temperature. Next, stop solution was added and absorbance was measured at 490 nm on a multi-well plate reader (Spectra Max iD3, Molecular Devices, San Jose, CA, USA). Cytotoxicity was determined as follows: cytotoxicity (%)=[(compound LDH activity – spontaneous LDH activity)/(maximum LDH activity – spontaneous LDH activity)] × 100. The maximum LDH activity was prepared by treating cells with lysis buffer. The medium used in the LDH assay contained 1% FBS (Gibco, Waltham, MA, USA). Three independent experiments were performed for each condition.

## 5. Conclusions

*S*(+)-(2*E*)-*N*-(2-hydroxypropyl)-3-phenylprop-2-enamide (compound KM-568) constitutes a cinnamamide derivative with configuration *S* in a chiral center and 2-hydroxypropyl substituent in the amide moiety. Its chemical structure and configuration were confirmed by NMR spectroscopy, elemental analysis, and crystallography. It proved beneficial anticonvulsant activity in several animal models of seizures and epilepsy, including those considered as models of refractory symptoms (i.e., 6-Hz 44 mA, LTG-resistant kindled rats, pilocarpine-induced status epilepticus model). Compound KM-568 was identified as a moderately stable derivative in an in vitro mouse liver microsome system. According to the Ames microplate format mutagenicity assay performed, we may conclude that KM-568 is not a base substitution or frameshift mutagen. Cytotoxicity evaluation in two cell lines (HepG2 and H9c2) proved the safety of the compound in concentrations up to 100 µM. The obtained results in terms of anticonvulsant activity and safety profile make it a good lead compound for further preclinical studies on novel treatment options for epilepsy. 

## 6. Patents

The title compound was a subject of our European Patent Claim: Gunia, A.; Waszkielewicz, A.M.; Marona, H. New alkanolamide derivatives of cinnamic acid and use of alkanolamide derivatives of cinnamic acid for preparation of drugs, PCT/PL2013/000139 [45].

## Figures and Tables

**Table 1:** Biological response in anticonvulsant and neurotoxicity evaluation in mice after intraperitoneal (*i.p*.) and oral (*p.o*.) administration of KM-568.

	Mice, *i.p.*			Mice, *p.o.*		
Test	KM-568 (mg/kg)	Results ^a^	ED_50_/TD_50_ (mg/kg) ^b^	KM-568 (mg/kg)	Results ^a^	ED_50_/TD_50_ (mg/kg) ^b^
MES	25	0/8	44.46 (42.50–47.93)	50	0/8	86.6 (74.48–102.11)
	38	1/8		60	2/8	
	41	0/8		70	2/8	
	42	2/8		90	3/8	
	44	5/8		120	7/8	
	50	7/8		150	8/8	
*sc*MET	70	2/8	104.29 (74.63–130.51)	50	1/8	107.27 (68.07–148.03)
	100	3/8		100	4/8	
	138	5/8		150	4/8	
	170	8/8		200	8/8	
6-Hz (32 mA)	50	1/8	71.55 (57.99–81.87)			
	75	3/8				
	87	7/8				
	110	8/8				
6-Hz (44 mA)	50	0/8	114.4 (81.75–138.57)			
	100	3/8				
	150	6/8				
	200	8/8				
Bicuculine	130	0/8	>130			
Picrotoxin	70	1/8	94.11 (73.85–113.62)			
	100	5/8				
	130	7/8				
TOX	100	0/8	148.47 (140.08–158.84)	200	0/8	344.14 (286.55–400.89)
	125	0/8		300	3/8	
	138	2/8		350	5/8	
	150	5/8		400	5/8	
	170	7/8		500	7/8	
	200	8/8				

^a^ Results presented as number of protected/tested animals in seizure models or number of animals displaying neurotoxicity/number of animals used in neurotoxicity assessment; ^b^ Calculated ED_50_ or TD_50_ value with 95% confidence interval in parentheses; the compound KM-568 was tested 0.25 h after administration (for 6-Hz 44 mA (*i.p*.), MES (*p.o*.) and *sc*MET (*p.o*.) the time was 0.5 h).

**Table 2:** Biological response in anticonvulsant and neurotoxicity evaluation in rats after intraperitoneal (*i.p*.) and oral (*p.o*.) administration of KM-568.

	Rats, *i.p*.			Rats, *p.o.*		
Test	KM-568 (mg/kg)	Results ^a^	ED_50_ or TD_50_ (mg/kg) ^b^	KM-568 (mg/kg)	Results ^a^	ED_50_ or TD_50_ (mg/kg) ^b^
MES	15	1/8	27.58 (18.84–41.74)	20.0	1/8	30.81 (24.16–37.13)
	22	3/8		30.0	3/8	
	30	5/8		35.0	5/8	
	60	7/8		40.0	7/8	
*sc*MET	30	1/8	41.72 (33.54–49.37)	62.5		>250
	45	4/8		125.0		
	60	8/8		250.0		
	95	8/8				
TOX	60	0/8	95.21 (79.8–110.42)	125.0	0/2	>500
	80	0/8		250.0	0/2	
	100	2/8		500.0	0/2	
	120	5/8				
	140	7/8				

^a^ Results presented as number of protected/tested animals in seizure models or number of animals displaying neurotoxicity/number of animals used in neurotoxicity assessment; ^b^ Calculated ED_50_ or TD_50_ value with 95% confidence interval in parentheses; the compound KM-568 was tested 0.25 h after administration for MES and *sc*MET (*i.p*.), 0.5 h for TOX (*i.p*.), 1.0 h for MES, *sc*MET and TOX (*p.o*.).

**Table 3:** Results of tests performed in Frings mice after 0.25 h of intraperitoneal administration of compound KM-568.

KM-568 (mg/kg)	Results ^a^	ED_50_ (mg/kg)
10.0	1/8	13.21 (11.22–15.11)
12.5	3/8	
15.0	6/8	
17.5	7/8	

^a^ Results presented as number of protected/tested animals.

**Table 4:** Results of intravenous metrazol seizure threshold (*iv*MET) test in mice performed after 0.25 h of *i.p.* administration of tested compound. Results are presented as mean ± SEM for ten animals for each dose.

KM-568 (mg/kg)	Time to twitch (sec)	Twitch * (mg/kg)	Time to clonus (sec)	Clonus * (mg/kg)
0	26.1 ± 1.7	26.8 ± 1.5	28.7 ± 1.8	29.6 ± 1.6
44	35.8 ± 1.5	37.8 ± 1.4	48.2 ± 3.5	50.8 ± 3.5
148	57.9 ± 1.6	61.3 ± 2.2	75.9 ± 2.9	80.2 ± 3.2

* Calculated metrazol dose causing first twitch and clonus.

**Table 5:** Results of mesial temporal lobe epilepsy model (MTLE) for KM-568 tested at 114 mg/kg (*i.p*.).

Recording Period	HPD * Counts	Mean HPD Counts	SEM	Effect (% of Baseline)
Baseline	16; 18; 21; 14	17.3	1.49	-
20–40 min	14; 8; 17; 12	12.8	1.89	73.9

* HPD - hippocampal paroxysmal discharges.

**Table 6:** Results of cornel kindling in mice after 0.5 h of *i.p.* administration of KM-568.

KM-568 (mg/kg)	Results ^a^	Individual Seizure Score ^b^	Average Seizure Score	ED_50_ (mg/kg)
57	1/8	4,5,5,0,4,5,4,5	4.0	79.17 (60.34–98.26)
84	5/8	4,5,3,3,3,3,3,5	3.625	
115	7/8	0,0,0,4,0,0,0,1	0.625	

^a^ Results presented as number of protected/tested animals, ^b^ according to the Racine scale.

**Table 7:** Results of a hippocampal kindled rat model after intraperitoneal administration of compound KM-568.

KM-568 (mg/kg)	Time (min)	Results ^a^	Seizure Score ^b^ ± SEM	Seizure Duration (sec) ± SEM	ED_50_ (mg/kg)
23	0		5.0 ± 0.0	66.83 ± 10.77	24.21 (19.4–26.54)
	15	2/6	3.67 ± 0.84	55.67 ± 6.44	
	45		5.0 ± 0.0	65.83 ± 14.61	
	75		5.0 ± 0.0	56.67 ± 6.96	
	100		5.0 ± 0.0	57.83 ± 9.27	
	135		5.0 ± 0.0	55.17 ± 9.48	
27	0		5.0 ± 0.0	56.20 ± 7.70	
	15	4/5	1.8 ± 0.97 *	50.4 ± 3.08	
	45		4.2 ± 0.80	80.0 ± 9.42 *	
	75		3.4 ± 0.98	70.6 ± 27.57	
	100		5.0 ± 0.0	62.5 ± 10.71	
	135		5.0 ± 0.0	95.0 ± 17.78 *	

^a^ Results presented as number protected/tested animals, ^b^ The mean value for five or six mice, the seizure score expressed in Racine scale, * data significantly different from control.

**Table 8:** Results of lamotrigine-resistant amygdala kindled rat model after 0.25 h of intraperitoneal administration of compound KM-568.

KM-568 (mg/kg)	Time of Test (h)	Seizure Score ± SEM	Seizure Duration (sec) ± SD	Results ^a^	ED_50_ (mg/kg)
control	0.0	5.0 ± 0.0	156.43 ± 7.31	0/7	58.69 (38.09–120.15)
20	0.25	5.0 ± 0.0	140 ± 17.55	0/7	
control	0.0	5.0 ± 0.0	118.86 ± 13.87	0/7	
40	0.25	3.86 ± 0.74	94.71 ± 8.72	2/7	
control	0.0	5.0 ± 0.0	86.71 ± 9.68	0/7	
80	0.25	2.86 ± 0.63 *	56.57 ± 12.34	5/7	

^a^ Results presented as number of protected/tested animals, protection defined as Racine score <3. * data significantly different from control.

**Table 9:** Results of pilocarpine-induced status epilepticus model in rats (compound KM-568 was administered *i.p*.).

Time (h)	KM-568 (mg/kg)	Results ^a^	ED_50_ (mg/kg)	ED_97_ (mg/kg)
0	200	8/8		
0.5	200	1/8	279.45 (212.61–344.62)	498.2 (378.3–1526.9)
	300	5/8		
	400	7/8		

^a^ Results presented as a number of protected/tested animals.

**Table 10:** Results of a formalin test performed in mice at a dose of 44 mg/kg (*i.p*.).

Phase	AUC ^a^				
	Methylcellulose	KM-568	% Control	SEM	*p* Value ^b^
Acute	241.17	184.18	76.37	13.03	>0.05
Inflammatory	819.31	556.81	67.96	13.27	>0.05

^a^ AUC-total area under curve in time after time of licking (time of formalin injection); ^b^ Student’s *t*-test.

**Table 11:** Results of mutagenicity assessment of KM-568 in the Ames microplate format (MPF) assay presented as fold induction of the number of positive wells over baseline ^a^.

Compound	Concentration (mM)	*Salmonella typhimurium*			
		TA98	TA100	TA1535	TA1537
KM-568	0.1 0.2 0.5	0.1 0.9 0.7	0.8 0.7 1.0	0.7 1.3 1.1	1.0 0.7 0.7
PC ^b^		6.4	3.4	14.7	48.0

^a^ Baseline = mean zero-dose control + 1 SD) ^b^ Positive controls: 2-nitrofluorene (2-NF) at 2 µg/mL (TA98); 4-nitroquinoline-*N*-oxide (4-NQO) at 0.1 µg/mL (TA100); *N*-4-aminocytidine (N4-ACT) at 100 µg/mL (TA1535); 9-aminoacridine (9-AAc) at 15 µg/mL (TA1537).
